# Fixation instability, astigmatism, and lack of stereopsis as factors impeding recovery of binocular balance in amblyopia following binocular therapy

**DOI:** 10.1038/s41598-022-13947-y

**Published:** 2022-06-20

**Authors:** Éva M. Bankó, Mirella Telles Salgueiro Barboni, Katalin Markó, Judit Körtvélyes, János Németh, Zoltán Zs. Nagy, Zoltán Vidnyánszky

**Affiliations:** 1 Brain Imaging Centre, Research Centre for Natural Sciences, Magyar tudósok körútja 2, Budapest, 1117 Hungary; 2 Department of Ophthalmology, Semmelweis University, Budapest, Hungary; 3 Bionic Innovation Center, Budapest, Hungary

**Keywords:** Paediatric research, Rehabilitation, Sensory processing, Developmental disorders, Striate cortex

## Abstract

Dichoptic therapy is a promising method for improving vision in pediatric and adult patients with amblyopia. However, a systematic understanding about changes in specific visual functions and substantial variation of effect among patients is lacking. Utilizing a novel stereoscopic augmented-reality based training program, 24 pediatric and 18 adult patients were trained for 20 h along a three-month time course with a one-month post-training follow-up for pediatric patients. Changes in stereopsis, distance and near visual acuity, and contrast sensitivity for amblyopic and fellow eyes were measured, and interocular differences were analyzed. To reveal what contributes to successful dichoptic therapy, ANCOVA models were used to analyze progress, considering clinical baseline parameters as covariates that are potential requirements for amblyopic recovery. Significant and lasting improvements have been achieved in stereoacuity, interocular near visual acuity, and interocular contrast sensitivity. Importantly, astigmatism, fixation instability, and lack of stereopsis were major limiting factors for visual acuity, stereoacuity, and contrast sensitivity recovery, respectively. The results demonstrate the feasibility of treatment-efficacy prediction in certain aspects of dichoptic amblyopia therapy. Furthermore, our findings may aid in developing personalized therapeutic protocols, capable of considering individual clinical status, to help clinicians in tailoring therapy to patient profiles for better outcome.

## Introduction

Amblyopia is diagnosed when distance best-corrected visual acuity (BCVA) is impaired, usually unilaterally, with no obvious organic changes in the visual pathways. Amblyopia is relatively frequent, affecting approximately 3% of the population^1,2^. It is also characterized by compromised contrast sensitivity binocular balance at mid and high spatial frequencies^3,4^, along with disturbed binocular functions^2,5–13^ accompanied by visuomotor disfunctions^14^, including abnormal fixational eye movements and/or eccentric fixation^15–18^, which seems to be a limiting factor for the restoration of binocular vision in amblyopic patients^19^.

Classical amblyopic treatments are based on the penalization of the dominant eye, which has been widely used to improve monocular visual acuity^20,21^. Unfortunately, however, occlusion therapy has a limited impact on binocular vision even when applied during early childhood^22^. Contrast sensitivity and binocular balance were consistently reported to remain deficient in amblyopic patients achieving normal visual acuity, therefore, considered treated^23–25^. To address the shortcomings of occlusion therapy, a diversity of experimental protocols using binocular approaches, aiming to reestablish binocular vision, have emerged in the last decade (for review see^2,26–28^). They have been successfully applied to improve visual functions in amblyopia by: (1) increasing visual acuity both in adult and pediatric patients^29–39^, (2) partially recovering binocular vision, resulting in better stereoacuity^38–43^, and (3) improving contrast sensitivity in adult amblyopic eyes^44–46^. These modern methods usually take advantage of dichoptic stimulation, often utilizing active or passive 3D technology^32,33,37,38,41,47^, but few of them create a three-dimensional environment^39,43^. Despite the overall promising results of dichoptic treatments, large inter-individual variation is observed^26,48^, similar to what has been observed in occlusion treatment.

Several limiting factors preventing amblyopic recovery in occlusion therapy are well established such as patients’ compliance^49–53^, poor initial visual acuity^49,52,54,55^, age at treatment onset^49,54^, high refractive error^52,55^. In addition, astigmatism of more than 1.5 diopters^49^, and type of amblyopia^51,52^ have also been implicated. Interestingly, fixation instability, relatively neglected in standard eye examination, has been associated with longer patching treatment^56–58^, poor stereopsis improvement, or lack thereof^57,58^, and a higher risk of amblyopic relapse^57^. Despite recent results^37,59,60^, a systematic understanding of limiting factors, especially for dichoptic approaches, is still lacking and these limitations might influence treatment efficacy.

By looking into changes in visual functions using multivariate analysis that considers all variables at the same time, we were able to study the quantitative contributions of clinical parameters to the success of dichoptic therapy using a new stereoscopic 3D, augmented reality (AR)-based binocular training program developed for children and applied both in pediatric and adult amblyopic populations. Our findings provide important insights about how baseline clinical status influences the efficacy of binocular treatment. A unified therapeutic model for treating amblyopia that is capable of considering individual clinical status is also proposed. It incorporates the present gold-standard treatment, augmented by emerging binocular therapeutic alternatives. Thus, our results contribute to previous efforts to customize amblyopic therapy in order to yield better treatment outcome^48^.

## Methods

### Participants

The tests were performed according to the tenets of the Declaration of Helsinki and were approved by the United Ethical Review Committee for Research in Psychology of Hungary (EPKEB; approval numbers 2014/5 and 2016/4). Participants were 45 amblyopic patients: 27 pediatric (mean age = 8.55 ± 2.12 years) and 18 adult (mean age = 34.09 ± 8.97 years) patients. Three children discontinued the training after 10 sessions and were removed from all analysis, leaving a total of 24 pediatric patients (mean age = 8.79 ± 2.13 years). Informed consent was obtained from all participants—and the legal guardians of pediatric patients—who underwent complete ophthalmic and orthoptic examinations at each follow-up examination. In the case of children, refractive error was measured under cycloplegia in a separate session. Inclusion criteria were as follows: ages of 5–13 years and 18–60 years for pediatric and adult patients, respectively, logMAR VA of 0.1–1.0 in the amblyopic, 0.1 or better in the dominant eye, with at least one line difference between eyes, heterotropia aligned with surgery or spectacle correction to within 10 prism diopters at near, absence of ophthalmological diseases, other than strabismic and/or anisometropic amblyopia, and the absence of neurological diseases that could affect the visual system. Our choice of including minimally amblyopic patients (i.e. one line difference between eyes; only 10% of our patient population according to the line assignment method) is contrary to common practice but it was to assist the prediction analysis with a wide range in depth of amblyopia. In addition, patients had to be past refractive adaptation (minimum of 4–6 weeks after new spectacle correction) and were not allowed to simultaneously participate in any other forms of amblyopia treatment other than the one under investigation. Thus, patching was also discontinued for the duration of the training.

Table 1 summarizes participants' information including etiology, refraction, baseline VA, and stereoacuity. Patients were assigned into etiological groups based on the following criteria: patients with ≥ 1 D difference across the most anisometropic meridian were categorized as anisometropic^61^; patients who had heterotropia on examination either at distance or near or had a history of strabismus were categorized as strabismic (S); while patients who were affected by both were categorized as mixed etiology (SA)^60,61^. For anisometropic patients, a distinction was made whether they had purely spherical anisometropia (A)—≥ 1 Dsph difference between eyes—or both spherical and astigmatic anisometropia (AA)—≥ 1 Dsph & ≥ 1 Dcyl difference between eyes^62^. Furthermore, astigmatism was also considered by itself: a patient was categorized as having astigmatism if the amblyopic eye had ≥ 0.75 cylinders regardless of the dominant eye’s refraction status.

**Table 1:** Patients' information.

Pediatric	Age/G	Refraction		Heterotropia/heterophoria angle	Fixation	BCVA (logMAR)		Interocular BCVA (logMAR)	Log stereo	Past therapy
		Right eye	Left eye			Right eye	Left eye			
AA1	7/F	+ 1.50 − 0.50 180°	+ 5.00 − 1.50 180°	D−, N7∆ EP		− 0.02	0.62	0.64	2.30	Occl
S1	9/M	+ 5.75 − 0.75 150°	+ 6.00	D8∆, N18∆ EP		0.12	0.20	0.08	2.60	Occl
A1	11/F	+ 7.00 − 0.75 150°	+ 1.50 − 0.50 40°	–		0.30	− 0.08	0.38	2.30	–
SA1	8/F	+ 5.00 − 0.25 3°	+ 6.00 − 0.25 5°	D12∆, N− XT^†^		0.00	0.36	0.36	4.00	Occl
AA2	10/F	+ 0.75 − 0.25 11°	+ 5.50 − 3.50 179°	D2∆, N− XP		0.00	0.30	0.30	2.60	–
AA3	10/M	+ 5.50 − 1.50 130°	+ 1.00	–		0.10	− 0.10	0.20	1.90	Occl
AA4	6/M	+ 5.00 − 2.25 180°	+ 3.00 − 1.00 170°	D = N2∆ XP		0.24	0.10	0.14	1.78	Occl
A2	11/F	+ 1.50	+ 6.25 − 0.75 30°	–		− 0.10	0.20	0.30	2.15	Occl
S2	9/M	+ 3.00 − 0.50 170°	+ 2.75 − 0.25 179°	D4∆, N9∆ ET^†^	Ecc	0.30	− 0.02	0.32	4.00	Occl
AA5	7/F	+ 4.25 − 3.25 15°	+ 1.75 − 0.25 3°	–		0.22	0.04	0.18	1.90	Occl
A3	6/M	+ 6.75 − 0.50 110°	+ 2.00 − 1.00 90°	–		0.26	0.02	0.24	1.90	Occl
A4	13/F	+ 6.25	+ 3.50	D2∆, N4∆ EP		0.34	− 0.10	0.44	2.30	Occl
A5*	7/F	− 1.25	− 7.00	–		− 0.10	0.50	0.60	2.15	Occl
SA2	12/M	+ 2.00 − 1.5 20°	+ 6.75 − 7.00 165°	–†		0.00	0.24	0.24	2.60	Occl
S3	10/F	+ 5.00	+ 5.50	D = N2∆ EP		− 0.02	0.44	0.46	3.55	Occl
A6	8/F	+ 3.50 − 3.75 6°	+ 4.25 − 3.50 13°	–		0.30	0.10	0.20	1.70	Occl
SA3	6/M	Plan − 2.00 15°	− 5.50 − 2.00 150°	D20∆, N8∆ XP		0.02	0.34	0.32	4.00	Occl
AA6	10/M	+ 7.75 − 0.75 20°	+ 8.00 − 1.00 135°	–		0.00	0.16	0.16	1.78	Occl
A7*	7/F	+ 1.25	+ 4.50	–		− 0.04	0.14	0.18	1.90	Occl
SA4	10/F	+ 4.00 − 1.00 20°	+ 6.50	D9∆, N10∆ ET		0.04	0.24	0.20	2.30	Occl
AA7*	6/M	− 1.00 − 1.50 12°	+ 0.75 − 0.25 15°	–		0.16	0.02	0.14	1.90	Occl
AA8	7/F	+ 1.75 − 1.25 165°	− 5.00 − 1.25 20°	–		0.14	0.66	0.52	2.90	Occl
S4	11/F	+ 1.75 − 1.75 3°	+ 2.00 − 1.75 5°	D− , N7∆ EP		0.20	0.00	0.20	2.30	–
A8	8/M	+ 3.75 − 0.25 36°	+ 6.00 − 0.50 174°	–		− 0.02	0.16	0.18	2.15	Occl
S5	10/M	+ 2.00 − 2.00 180°	+ 1.50 − 1.50 180°	D4∆, N7∆ EP		0.18	0.06	0.12	2.60	Occl
AA9	5/M	+ 2.00 − 0.50 10°	+ 5.00 − 3.50 165°	–		0.04	0.34	0.30	1.70	–
SA5	7/M	− 3.00 − 1.50 70°	+ 0.75 − 0.75 90°	D− , N10∆ EP		0.48	0.10	0.38	4.00	Occl
*mean*	8.55									
*sd*	2.12									
**Adult**										
SA6	38/F	+ 0.50 − 0.75 170° (5∆ base-out)	− 0.50 − 0.75 35° (5∆ base-out)	D = N7∆ ET	Ecc	0.86	0.04	0.82	4.00	Occl
S7	44/F	+ 3.50	+ 3.25	D−, N6∆ ET		0.08	0.00	0.08	4.00	Occl
AA10	38/M	− 4.25 − 3.00 150°	− 2.50	–		0.32	− 0.18	0.50	1.60	–
SA7	46/M	+ 2.00	+ 4.50 − 4.00 24°	D4∆, N6∆ ET		− 0.12	0.50	0.62	4.00	Occl
A9	47/F	+ 4.50 (add + 0.75)	+ 3.75 (add + 0.75)	–		0.46	− 0.24	0.70	2.60	–
A10	39/F	− 0.50 − 0.25 141°	+ 3.50 − 0.75 43°	–		− 0.16	0.54	0.70	2.30	–
SA8	40/M	− 7.75 − 3.75 175°	− 3.00 − 0.50 25°	D = N12∆ XP		0.46	− 0.20	0.66	1.60	Occl
S8	29/M	− 0.25 − 0.25 111°	− 0.75 − 0.50 89°	D8∆, N2∆ EP^†^		0.64	− 0.06	0.70	2.30	Occl
AA11	29/M	− 0.25	+ 5.25 − 2.75 61°	–		− 0.10	0.40	0.50	2.15	Occl
S9	34/M	+ 1.00 (4∆ base-out)	+ 1.25 (4∆ base-out)	D = N10∆/2∆, ET/HypoT		− 0.26	0.94	1.20	4.00	Occl
S10	34/F	− 0.75 − 0.50 96°	− 0.75 − 1.25 74°	–		− 0.10	0.20	0.30	2.60	–
A11	27/F	+ 4.50 − 1.50 180°	− 0.50 − 0.75 152°	–		0.42	− 0.16	0.58	4.00	–
S11	46/M	Plan − 1.25 110° (add + 1.25)	+ 0.25 − 1.50 75° (add + 1.25)	–	Ecc	0.02	0.50	0.48	2.60	Occl
A12	31/M	+ 4.50 − 1.00 180°	+ 3.25 − 0.75 30°	–		0.64	0.04	0.60	3.55	Occl
AA12	26/M	Plan	+ 5.00 − 1.00 175°	–		− 0.22	0.90	1.12	4.00	–
AA13	19/F	− 0.50 − 1.50 175°	− 5.75 − 3.00 20°	–		0.00	0.20	0.20	1.70	Occl
SA9	28/M	+ 1.00 − 0.50 93°	+ 4.00 − 0.75 71°	D = N6∆ ET	Ecc	− 0.16	0.76	0.92	4.00	Occl
A14	43/M	− 0.50 − 0.50 160°	+ 2.50	–		− 0.08	0.48	0.56	2.30	–
*mean*	34.09									
*sd*	8.97									

*A* spherical anisometropia, *AA* spherical and astigmatic anisometropia, *S* strabismus, *SA* mixed amblyopia, *G* gender, *F* female, *M* male, *BCVA* best-corrected visual acuity, *D* distance, *N* near, *ET* esotropia, *XT* exotropia, *EP* esophoria, *XP* exophoria, *Ecc* eccentric fixation, *Occl* occlusion therapy.

*Patients dropped out from the therapy.

^†^Patients undergone strabismus corrective surgery.

All participating subjects underwent binocular treatment of 20 h of game-play within a three-month period and underwent examinations to test visual functions: best-corrected visual acuity at near and distance (nVA & dVA), spatial contrast sensitivity (CS) function, and stereoacuity both before treatment (baseline visit: V_BL_) and after 20 sessions (V_20h_). In addition, pediatric patients were evaluated two additional times during the training period: after 10 sessions (V_10h_), and one month after treatment was discontinued (follow-up visit: V_FU_) to monitor changes more closely as occlusion therapy for children was suspended throughout the entire study (i.e. up until V_FU_). Patients were instructed to play at least twice a week with a maximum break of three days. Children attended training sessions in the research institute, while half of the adults received the set-up for home-training with online progress monitoring.

### Visual functions

Best-corrected distance and near visual acuity (BCVA) was measured using crowded tumbling E logMAR visual acuity charts for both children and adults. Tumbling E Series ETDRS chart (Precision Vision Ltd., La Salle, IL, USA) was used for distance vision measurement, and was viewed from 4 m using the corresponding backlit illumination cabinet at a luminance of 500 cd/m^2^. Tumbling E Runge Pocket Near Vision Test Card (Precision Vision Ltd., La Salle, IL, USA) was used for near vision measurement, and was viewed from 40 cm. A forced-choice testing method was applied, and visual acuity was scored using the standard technique of subtracting 0.02 logMAR units for each correctly identified optotype.

Contrast sensitivity function was measured using a standard lit Sine Wave Contrast Sensitivity Chart (Stereo Optical Company Inc., Chicago, IL, USA) with 3 m of viewing distance. The test comprised eight columns of tilted Gabor patches with decreasing contrast at 1.5, 3, 6, 12 and 18 cycles-per-degree (cpd). Patients’ task was to indicate the tilting direction of a given patch (leftward, vertical, or rightward tilt), therefore, corresponding to a three-alternative forced choice (3AFC) task. The test began with the upper most row corresponding to 1.5 cpd in a decreasing contrast order until they made an incorrect choice. If patients gave an incorrect response, the preceding (i.e. higher) contrast value was retested. The same procedure was repeated at each spatial frequency. Contrast sensitivity was the inverse of the contrast at the last correctly identified patch. For characterizing changes in contrast sensitivity, a broad contrast sensitivity metric, the area under the log contrast sensitivity function (AULCSF) was used^63,64^. This was calculated by fitting a third-order polynomial to the log contrast sensitivity versus log spatial frequency data of each subject and integrating between the lowest and highest spatial frequencies^65^.

Stereopsis was measured for near distance (40 cm) using the Titmus graded circles stereo test as in the Stereo Fly Test (Stereo Optical Company Inc., Chicago, IL, USA). It shows graded circles containing nine panels with stimulus disparity spanning from 800 to 40 arc seconds. Each panel contained four contoured circles, of which only one had a crossed disparity. Subjects wearing polarized glasses were asked to identify the circle that appeared to pop out of the plane (4-AFC task). The test began with the panel containing the largest disparity and going in order with the next panel until the patient made an incorrect choice. If there was an incorrect response, the preceding panel was retested. In case subjects could not identify the stimulus with highest disparity (800″), the stereo fly diagram was shown and pinching of the fly wings was required to achieve 3500 arc seconds. If they succeeded, this value was entered as their stereoacuity, otherwise they were assigned 10,000 arc seconds (log stereoacuity of 4) corresponding to nil stereoacuity. For statistical analysis, the log transformed values were used.

Macular sensitivity and fixation stability were measured in adult patients using the Expert Protocol of the Macular Integrity Assessment (MAIA; CenterVue, Padova, Italy) microperimeter. Pupillary dilatation was not used. The patient’s task was, as with conventional perimeters, to press a button to indicate the presence of a light spot whenever it was detected. The expert protocol allowed recording macular sensitivity at 37 macular points up to 10° of central visual field using Goldmann-standardized stimuli. In addition, the microperimeter, equipped with a scanning laser ophthalmoscope with real-time eye tracking system, provided fixation information with a sampling rate of 25 Hz. Only fixation stability obtained during the initial fixation phase of the microperimetric measurements is presented, which lasted 10 s with only the fixation point present. Due to the difficulty in obtaining microperimetric data in younger patients, a different fixation paradigm was employed for children using an IView X binocular infrared eyetracker (SensoMotoric Instruments GmBH, Teltow, Germany) with a sampling rate of 350 Hz. Each children underwent a separate fixation session prior to the binocular treatment, where they were required to fixate a 3° cross for 10 s. This procedure was repeated 10 times and out of the reliable trials, where children attended to the fixation point throughout the entire trial, only the one with the best overall fixation (smallest dispersion measure averaged across eyes) was used to facilitate comparison with adult measurements. Blinks and large saccades exceeding 1.5° were removed from the raw fixation data. Then the data were demeaned: the average of all fixation coordinates were subtracted from the data for each trail to evaluate the relative dispersion. This compensates for any possible shift during the measurement. For each eye position measurement (i.e., a pair of [x,y] coordinates), geometrical distance from the fixation point was calculated. The median distance was used as a measure of fixation stability in each subject separately for each eye with higher distance values meaning less stable fixation^66,67^. In addition, we have also calculated to more standard bivariate contour ellipse area (BCEA), which however suffers from normality assumptions and assumes fixation data is elliptically distributed, as opposed to the assumption free distance calculation^67^. Calculation of BCEA and the results obtained with it can be found in the “Supplementary Methods and Results”. To make the two types of measurements from the groups comparable, an interocular difference was calculated, and this was used as a predictor in the analyses along with factor ‘group’ to account for any remaining difference between the measurement types.

### Binocular treatment

**Figure 1:**
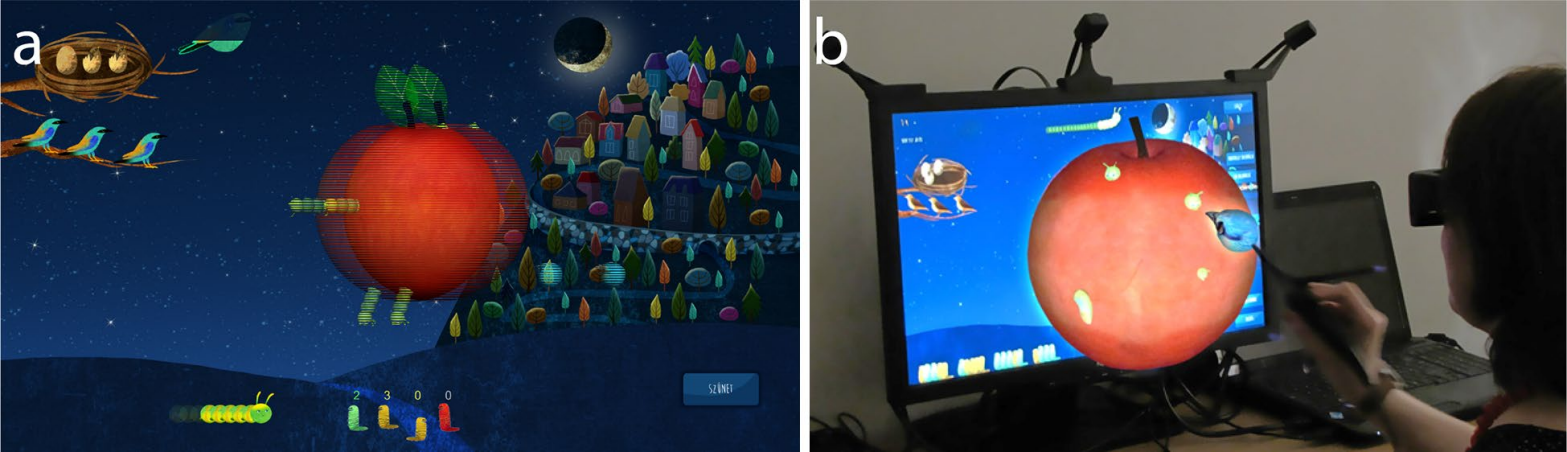
Stereopia. (**a**) Screenshot of the interactive game. (**b**) Perceived image during game play. The apple, the caterpillars (target) and the bird head (targeting object), which sits virtually at the tip of the 3D mouse, are perceived in 3D in front of the background, while the 3D mouse is held in hand. All 3D objects are part of an augmented reality, thus are intermixed with real objects (i.e. the patient’s hand), the percept adjusting upon change in perspective.

The treatment comprised of a playful software titled Stereopia in a fully immersive stereo 3D augmented reality environment built on the Leonar3Do equipment (Leonar3Do International Inc., Budapest, Hungary), which enables both viewing virtual 3D images on a passive 3D display and their manipulation in real time with a handheld 3D mouse. The display was an LG D2342 3D capable monitor with 1920 × 1080 resolution and interleaved 3D presentation, viewed through LG polarized glasses and the patients’ optical correction. Stereopia software, developed in collaboration with Leopoly Ltd. (Budapest, Hungary), uses an interactive stereo 3D approach to directly train stereovision and eye-hand coordination, as both the virtual image and the real manipulating hand can be seen at the same time. The program involves a highly engaging children’s video game to capture young patients' attention. The goal of the game was to capture 3D caterpillars emerging from a round fruit with a bird head sitting virtual at the tip of the 3D mouse. Players had to orient the mouse such that it was parallel with the caterpillar’s motion vector and was also at the right depth (Fig. 1). Amblyopic and fellow eyes viewed stereo counterparts of the same image on a dark background. If the patient did not see a stereoscopic percept during game play, the luminance—and as a result the contrast—of the fellow eye was decreased until the stereo percept was obtained. The game had four levels, while game speed was manipulated throughout the game based on performance using a 2 up/1 down staircase procedure. Luminance of the fellow eye and depth threshold was also assessed at the start of each session using built-in tests measuring suppression and stereoacuity with the gaming parameters set respectively. Children’s attention and motivation to play the game was high throughout the training. Most adults also enjoyed the game and found it challenging. Their main motivation, however, was the ability to finally do something to counter amblyopia.

### Statistical analysis

Monocular data obtained from each eye were used to calculate interocular values by subtracting the smaller value from the higher one (i.e. amblyopic eye (AE)–fellow eye (FE) for acuity and FE–AE for CS). Thus, higher interocular values indicated a bigger difference between eyes. Shapiro–Wilk’s test was used to verify normal distribution. In the case of paired comparisons of post- vs. pre-treatment and post-treatment vs. follow-up values, the distributions of the differences were verified. If they met the criteria of a normal distribution at 1% significance level, post- vs. pre-treatment data were analyzed via Student’s t-test. Otherwise, a nonparametric Wilcoxon matched pairs test was used. For group comparison, post-intervention results were calculated as change from baseline (CFB)—calculated as post–pre for CS of AE&FE, where an increase in value indicated improvement, and as pre-post for stereoacuity, logMAR VA of AE&FE, and for all interocular differences, where a decrease in value indicated improvement—separately for each eye and for interocular values. Thus, positive CFB values indicated improvement and negative values decrease as a result of training. These values were then entered into two-sample t-tests, or nonparametric Mann–Whitney U tests, in case the data were not distributed normally. Bonferroni correction was used to correct for multiple comparisons, separately for monocular (six different measures, p_Bonf_ < 0.05 corresponds to p < 0.008) and for interocular values (three different measures, p_Bonf_ < 0.05 corresponds to p < 0.016).

To analyze the treatment time course effects, data from the baseline visit and visits following 10 and 20 h of treatment (V_BL_, V_10h_, V_20h_) were entered into general linear mixed models, with ‘subject’ as a random factor, ‘time’ as a continuous [0, 1, 2] fixed factor, and their interaction to account for individual differences in the slope of the improvement in the model. This model, when ‘time’ was significant, indicated a gradual linear change in the visual functions as an effect of the treatment proportional to treatment time. Stereoacuity, interocular distance (dVA) and near (nVA) visual acuity, and interocular contrast sensitivity were analyzed. For each measured visual function, the distribution of the pooled data from all three visits were evaluated in terms of normality and were transformed into normal distributions using square-root transformation in case normality was not met. The transformation was performed by shifting the minimum of the distribution close to zero, where applicable, and taking the square root of each value. In the case of interocular near logMAR VA values, another square root transformation was necessary.

For modelling treatment outcome, general linear models (GLM) were used aided by multiple regression analyses to identify the important predictor variables. Change-from-baseline (CFB) stereoacuity and interocular distance (dVA), near visual acuity (nVA) and contrast sensitivity (CS) measures were used as dependent variables. (Monocular amblyopic measures were also analyzed, the results of which can be found in the “Supplementary Results”.) For possible predictors, the following variables were used: baseline measurements of stereoacuity and interocular values of dVA, nVA, and CS log transformed for normality, interocular fixation stability, and cylindrical diopter as continuous variables (i.e. covariates), and group (children vs. adults), age-group (< 9 years, 10–19 years, 20–39 years, or > 40 years), etiology (A, AA, S, or SA), heterotropia (present vs. not at V_BL_), sightedness (myopic vs. hyperopic), presence of astigmatism (≥ 0.75 Dcyl in the AE), orientation of astigmatic axis (WTR, ATR, OBL or none), past occlusion (ever occluded vs. not occluded), the presence of stereopsis at baseline, and post-treatment poor dVA (≥ 0.4 logMAR) in the amblyopic eye (the latter only in the case of contrast sensitivity) as categorical factors. As a first step, all of these predictors were entered into a multiple regression analysis with a forward stepwise approach separately for each dependent variable, which automatically eliminated the predictors that did not influence the respective dependent variable and kept only those that explained significant variance in the dependent variable. Next, these automatically selected predictors were entered into a GLM analysis (ANCOVA), where also interactions were considered between predictors based on visual inspection of the data and common sense. For each dependent variable, several models were created in an iterative manner aiming for maximizing model fit (adjusted R^2 ^), while minimizing residual error and the number of variables used to explain the data. The most economical model was chosen as the final model.

## Results

Compliance was high in both groups. By the end of the three months, 24 (89%) children have completed ≥ 75% of the required training, while 3 (11%) dropped out of the training after 10 sessions and their results could not be analyzed. Out of the 24 children following through with the training, 19 (79%) has completed all 20 sessions, the remaining 5 (17%) completing ≥ 90% of the required sessions. In adults, half of whose learning was only remotely supervised, all 18 (100%) completed ≥ 75% of the required training sessions within three months, 15 (83%) of them completing all 20 sessions.

Besides stereovision, the training effects measured monocularly were evaluated separately for each eye as well as calculating interocular values. Results pertaining to amblyopic and fellow eye monocular functions can be found in the “Supplementary Results”.

### Effects of the training on visual functions

**Figure 2:**
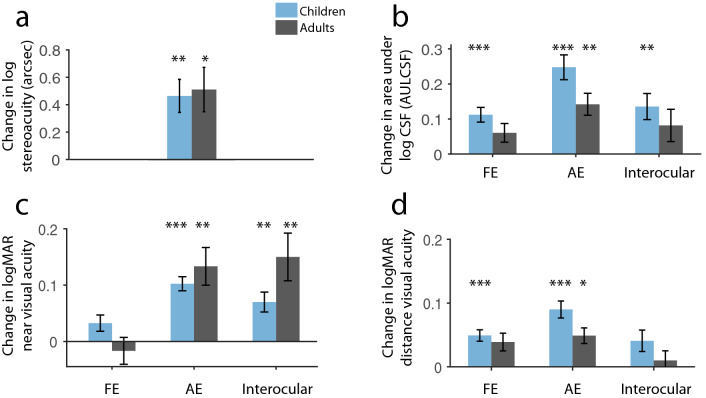
Average improvement in all measured visual functions. Change in log stereoacuity (**a**), contrast sensitivity (**b**), near logMAR visual acuity (**c**), and distance logMAR visual acuity (**d**) in children (blue) and adults (gray). *FE* fellow eye, *AE* amblyopic eye, N_Ch_ = 24, N_Ad_ = 18, Means ± SEM. *p_Bonf_ < 0.05, **p_Bonf_ < 0.01. ***p_Bonf_ < 0.001.

**Figure 3:**
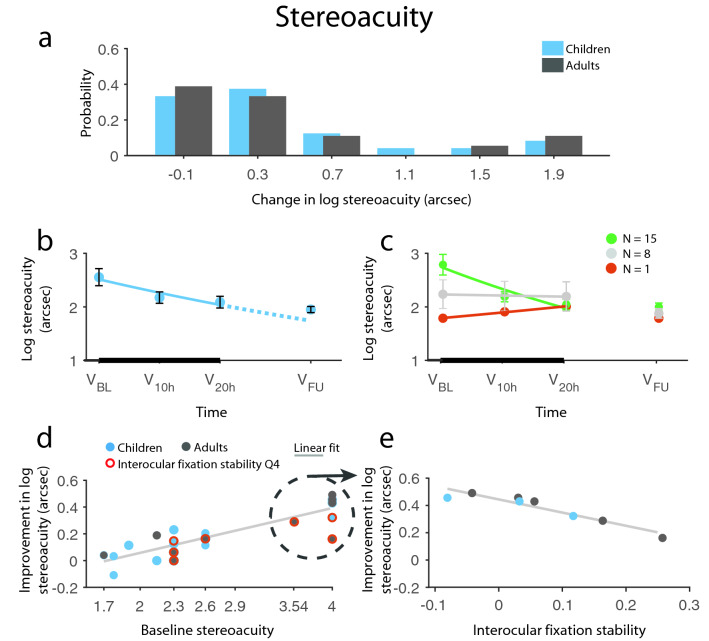
Improvement in stereoacuity. (**a**) Distribution of change from baseline (CFB) in log stereoacuity in children and adults. (**b**) Time course of stereacuity recovery in children. The negative exponential fit indicates improvement (solid line) during the therapy (thick black line) and the expected improvement rate (dotted line) had the therapy been continued. Children’s improvement remained stable at 1-m follow-up without regression. (**c**) Division of children’s change over time based on the exponent of the fitted exponential: green, red, and gray indicates improvement, decline, and no change, respectively. Children showing stereoacuity decline during the treatment regained their baseline stereoacuity at follow-up. (**d**, **e**) Results of the prediction analysis: (**d**) Patients’ improvement depended on their baseline steroacuity: bigger improvement was possible with poorer initial stereoacuity. (**e**) Improvement of stereoblind or patients with crude stereoacuity (≥ 3500″) strongly depended on their relative fixation stability: the more stable the fixation was, the more patients improved. Red circles signify patients, whose relative fixation stability falls in the worst quarter (Q4), light gray line indicates a linear fit. N_Ch_ = 24, N_Ad_ = 18, except for (**d**, **e**), where N_Ch_ = 19, N_Ad_ = 13. Means ± SEM.

**Figure 4:**
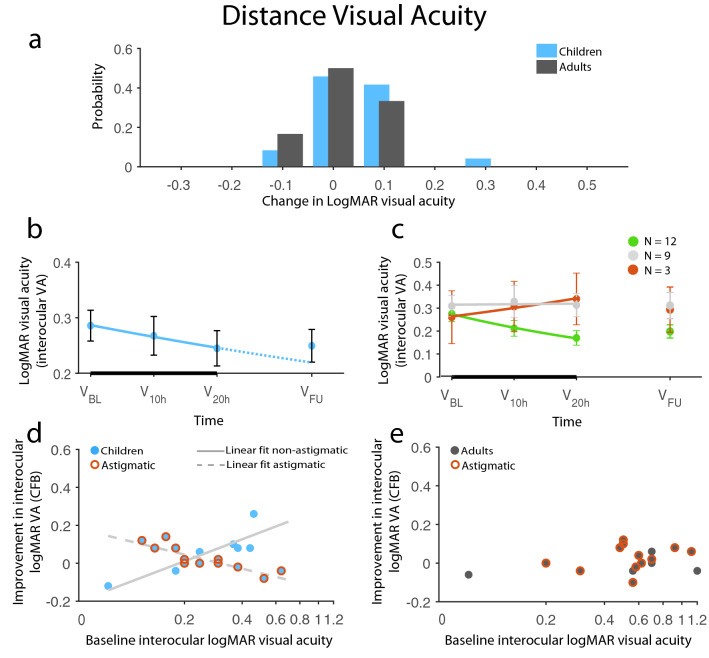
Improvement in distance logMAR visual acuity. (**a**) Distribution of change from baseline (CFB) in logMAR dVA in children and adults. (**b**) Time course of dVA recovery in children. The negative exponential fit indicates improvement (solid line) during the therapy (thick black line) and the expected improvement rate (dotted line) had the therapy been continued. Children’s improvement remained stable at 1-m follow-up without regression. (**c**) Division of children’s change over time based on the exponent of the fitted exponential: green, red, and gray indicates improvement, decline, and no change, respectively. (**d**, **e**) Results of the prediction analysis: (**d**) Children’s interocular dVA recovery was strikingly dependent on the presence of astigmatism: non-astigmatic children’s potential for improvement increased with worse baseline interocular dVA, while the opposite was true for astigmatic children. (**e**) Adults’ modest progress, on the other hand, did not depend on their baseline dVA, nor did the presence of astigmatism had any impact on it. Red circles signify astigmatic patients, light gray solid line indicates a linear fit for non-astigmatic children, while the dashed line is the linear fit for astigmatic children. N_Ch_ = 24, N_Ad_ = 18, except for (**d**, **e**), where N_Ch_ = 21, N_Ad_ = 17. Means ± SEM.

**Figure 5:**
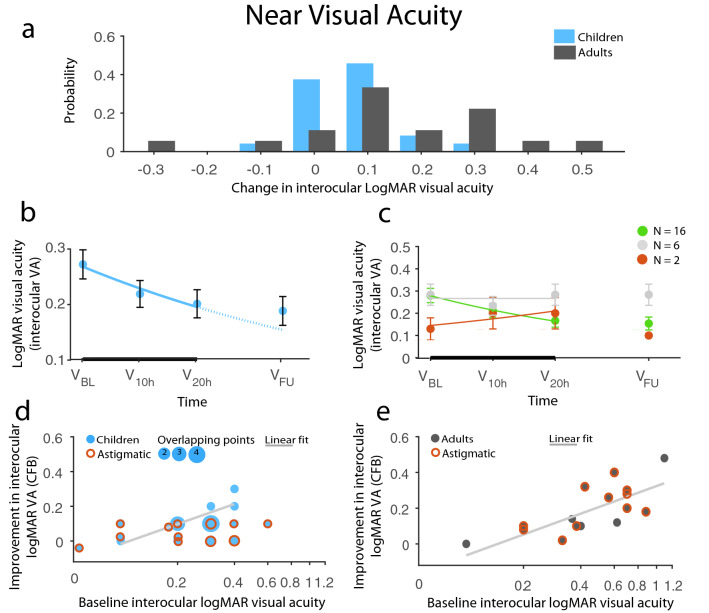
Improvement in near logMAR visual acuity. (**a**) Distribution of change from baseline (CFB) in logMAR nVA in children and adults. (**b**) Time course of nVA recovery in children. The negative exponential fit indicates improvement (solid line) during the therapy (thick black line) and the expected improvement rate (dotted line) had the therapy been continued. Children’s improvement remained stable at 1-m follow-up without regression. (**c**) Division of children’s change over time based on the exponent of the fitted exponential: green, red, and gray indicates improvement, decline, and no change, respectively. Children showing nVA decline during the treatment regained their baseline nVA at follow-up. (d-e) Results of the prediction analysis: (**d**) Children’s interocular nVA recovery was strongly limited by the presence of astigmatism: non-astigmatic children’s potential for improvement increased with worse baseline interocular dVA, while astigmatic children did not show improvement regardless of their baseline value. (**e**) Adults’ progress, on the other hand, was not impacted by astigmatism, but solely depended on their baseline nVA. Red circles signify astigmatic patients, light gray solid line indicates a linear fit for non-astigmatic children, while the dashed line is the linear fit for astigmatic children. The size of data points and red circles corresponds to the number of overlapping data points. N_Ch_ = 24, N_Ad_ = 18, except for (**d**, **e**), where N_Ch_ = 23, N_Ad_ = 16. Means ± SEM.

**Figure 6:**
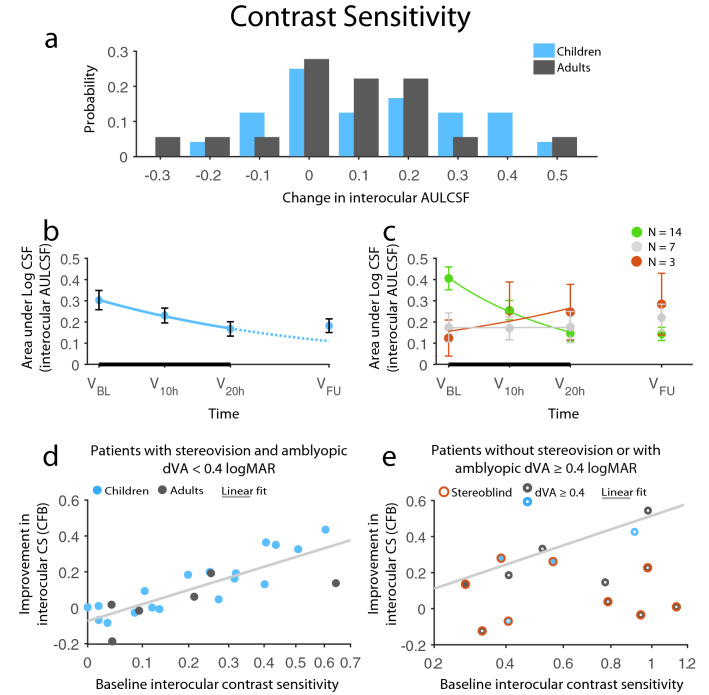
Improvement in AULCSF (Area Under Log Contrast Sensitivity Function). (**a**) Distribution of change from baseline (CFB) in AULCSF in children and adults. (**b**) Time course of contrast sensitivity recovery in children. The negative exponential fit indicates improvement (solid line) during the therapy (thick black line) and the expected improvement rate (dotted line) had the therapy been continued. Children’s improvement remained stable at 1-m follow-up without regression. (**c**) Division of children’s change over time based on the exponent of the fitted exponential: green, red, and gray indicates improvement, decline, and no change, respectively. (**d**, **e**) Results of the prediction analysis: (**d**) Patients’ improvement had a definitive dependence on their baseline contrast sensitivity: patients improved almost as much as there was room for improvement, given that they did not have either of two limiting factors: initial stereoblindness or acuity equal to or below a critical visual acuity of 0.4 logMAR. (**e**) The improvement of patients with only one of the limiting factors also lie along the same linear fit calculated for patients without either of the limiting factors, whereas patient affected by both factors showed no or very limited improvement. Red circles signify initially stereoblind patients, white dots in the center of datapoints mark acuity equal to or below 0.4 logMAR, while a light gray line indicates a linear fit for patients without limiting factors, which is copied to (**e**) as well. N_Ch_ = 24, N_Ad_ = 18, except for (**d**, **e**), where N_Ch_ = 22, N_Ad_ = 16. Means ± SEM.

Analyzing the pediatric population, significant improvement was found in stereoacuity (Fig. 2a; Wilcoxon matched pairs test: Z = 3.66, *p* = 0.0002), while the improvements were significantly stronger for the amblyopic compared with the fellow eye in the cases of near VA (nVA) and contrast sensitivity (CS), as their interocular difference showed a significant decrease as a result of the training (Fig. 2b,c; Z = 3.02, *p* = 0.0025 and paired t-test t_(23)_ = 3.64, *p* = 0.0014, respectively). In the case of distance VA (dVA), however, the decrease was less pronounced and failed to reach the specified Bonferroni-corrected significance threshold (Fig. 2d; p < 0.05/3 = 0.016; t_(23)_ = 2.43, *p* = 0.023). Looking at the adult population, the change was significant in the cases of stereoacuity and nVA (Z = 3.08, *p* = 0.0021 and t_(17)_ = − 3.54, *p* = 0.0025, respectively), but not in the cases of dVA and CS (t_(17)_ = 1.32, *p* = 0.20 and t_(17)_ = 1.76, *p* = 0.096 for dVA and CS, respectively), because of similar improvements in both amblyopic and fellow eyes. When compared directly, however, the two groups similarly benefited from the treatment as the change from baseline (CFB) in stereoacuity (two-samples t-test: t_(40)_ = − 0.24, *p* = 0.81) and in interocular values were comparable between groups (Figs. 3a, 4a, 5a, 6a; p < 0.05/3 = 0.016; p t_(40)_ = − 1.11, *p* = 0.27, Mann–Whitney U-test (n1 = 24, n2 = 18): Z = 2.16, *p* = 0.031 and t_(40)_ = − 0.91, *p* = 0.36, for stereoacuity, dVA, nVA and CS, respectively).

### Time course and stability of improvements in children

In the pediatric population with additional visits (visits following 10 h of treatment: V_10h_ and a 1-month follow-up: V_FU_; Figs. 3b, 4b, 5b, 6b), the hypothesis of gradual and/or linear improvements during training (V_BL_, V_10h_, V_20h_) was confirmed using a general linear mixed-model with elapsed treatment time as a continuous factor. There were significant linear improvements with time in stereoacuity (main effect of time: F_(1,24)_ = 19.32, *p* = 0.0002), nVA (F_(1,24)_ = 15.78, *p* = 0.0006), and CS (F_(1,24)_ = 14.30, *p* = 0.0010), while gradual change over time in dVA failed to reach the Bonferroni-corrected significance threshold (F_(1,24)_ = 6.19, *p* = 0.021). The pediatric patients were further divided into subgroups based on the tendency of change in their interocular values over time: to those patients improving, worsening, and stagnating based on the exponent value of their exponential fit being negative, positive, and close to zero, respectively (Figs. 3c, 4c, 5c, 6c). Approximately 60% of children showed improvements, ~ 30% did not change during the therapy, and ~ 10% declined. Nevertheless, the latter few mostly stabilized at their baseline value at V_FU_.

The stability of improvements was also investigated with results obtained one month after the cessation of the training, when children were instructed to refrain from occlusion, thus, they did not receive any treatment. Three patients were lost to follow-up, whereas one patient resumed occlusion therapy in this period. Hence, her data were excluded. Evaluating the remaining subjects, we observed stable improvements in visual functions from V_20h_ to V_FU_ in the cases of stereoacuity (Wilcoxon matched pairs test: Z = 1.43, *p* = 0.15), nVA and CS (Z = 1.21, *p* = 0.22, t_(19)_ = − 0.29, *p* = 0.77, respectively). Interocular dVA also did not change in the follow-up period (t_(19)_ = − 0.68, *p* = 0.50).

### Factors predicting improvement

To investigate the possible contribution of clinical factors to the individual variation in improvements observed for certain visual functions, an ANCOVA approach was used, which was validated using multiple regression analysis. We looked at baseline values from specific visual functions alongside the optical and etiological parameters and the baseline values of other measures to find out which affect therapy outcome, i.e. the change from baseline in the respective visual functions. Besides stereopsis, we focused on interocular values as a means of normalizing the treatment effect, thus reducing noise in the data. Nevertheless, similar models with monocular changes of the amblyopic eyes are reported in the “Supplementary Results”.

#### Stereorecovery is only possible with stable fixation

The best prediction model for stereoacuity changes, with a model fit of R_multiple_ = 0.90 and adjusted R^2^ = 0.79 (F_(3,28)_ = 40.27, *p* < 0.0001) explaining 79% of the variance of the data, was obtained. The final and most economical model included ‘baseline stereoacuity’, ‘baseline relative fixation stability’, and the interaction between these variables as predictors. Patients’ data, whose improvements were out of the measurement range (40″–3500″), were excluded, because their improvements, if any, could not be quantified. Four additional patients did not have fixation data, therefore, 32 patients were included in this prediction model.

Baseline stereoacuity had the strongest effect on stereoacuity improvement (main effect: F_(1,28)_ = 120.48, *p* < 0.0001): the worse the stereoacuity was for a given patient, the more that patient could improve, indicating that the training strongly modulated stereoacuity (Fig. 3d). Importantly, relative baseline fixation stability had a significant effect on therapy outcome (main effect: F_(1,28)_ = 7.87, *p* = 0.0090) as well as a significant interaction with baseline stereoacuity (F_(1,28)_ = 9.67, *p* = 0.0043): patients with better interocular fixation stability (i.e. smaller difference in fixation between the eyes) had a higher potential for stereoacuity improvement. However, as the interaction indicated, this effect was dependent on baseline stereoacuity. In fact, interocular fixation stability was a strong predictor for patients with only coarse or nil stereoacuity (≥ 3500″). The same pattern—alas with a slightly lesser goodness of fit—was present if fixation stability was calculated using the more conventional method of bivariate contour ellipse area (BCEA; see “Supplementary Results”). This was further supported by the following analyses: (1) partial correlation between stereoacuity changes and interocular baseline fixation stability—controlling for the effect of baseline stereoacuity—showed a highly significant effect for patient group with coarse or nil stereoacuity (r_partial_ = 0.94, *p* = 0.002, N = 8), but failed entirely when this group was excluded (r_partial_ = 0.075, *p* = 0.73, N = 24) ii) there was a significant positive correlation between fixation stability and stereoacuity improvements for the coarse or nil stereoacuity group (Fig. 3e; Spearman rho = 0.95, *p* < 0.0001, N = 8), (3) out of the three patients, excluded from this analysis because of non-measurable stereoacuity before and after the intervention, one with good fixation stability starting from suppression managed to achieve fusion by the end of the training, while the other two with poor fixation stability (within the worst 10%) did not progress from fusion to stereovision. Thus, the overall conclusion is that contrary to common expectation, poor stereopsis, or lack thereof, is not at all a contraindication for binocular treatment approaches. Rather, for patients lacking stereovision (outside of the Titmus stereo test range), a relatively stable fixation in the amblyopic eye is required for stereoacuity improvements using the 3D therapy reported here.

Even though the fixation measurements in the two groups were different, the inclusion of either ‘group’ or ‘age-group’ (either case: *p*s > 0.6) did not change the fit of the model, thus, possible differences between groups were not accounted for in the above model. Nevertheless, we have included separate analyses for each group. Despite the low number of data points, there was a similar pattern in the case of adult patients, where there was a significant interaction between baseline stereoacuity and relative fixation stability (F_(1,9)_ = 5.65, *p* = 0.041) as well as a significant main effect of baseline stereoacuity (F_(1,28)_ = 112.51, *p* < 0.0001). Importantly, this model had a very high adjusted R^2^ value (0.92), demonstrating that the interplay between baseline stereoacuity and relative fixation stability is enough to explain the observed gain in stereoacuity in the case of adults. On the other hand, the interaction was not significant in the case of children (F_(1,15)_ = 1.47, *p* = 0.24), even though the same pattern of correlation were observable for the three children with coarse or nil stereoacuity (Fig. 3e). The discrepancy between groups could, however, result from children’s lesser quality of fixation data or the fact that there were too few children with coarse or nil stereoacuity in our dataset.

#### Visual acuity improvement is limited by astigmatism in children

##### Interocular distance visual acuity

The final prediction model fit for dVA, with R_multiple_ = 0.91 and adjusted R^2^ = 0.75 (F_(12,25)_ = 10.32, *p* < 0.0001) explaining 75% of data variance, included the full factorial model of {‘baseline interocular distance visual acuity (dVA)’, ‘group’, and ‘presence of astigmatism’}, and ‘baseline interocular near visual acuity (nVA)’, ‘etiology’, and ‘past occlusion’ as predictors. Four patients were classified as outliers (i.e., standard residual ≥ 2 SD), and were removed, therefore the final prediction model included 38 patients. As expected, baseline dVA had a significant, although not the most pronounced effect on therapy outcome (main effect: F_(1,25)_ = 23.64, *p* < 0.0001). This could not be attributed to a simple tendency of patients with higher interocular difference in dVA being able to improve more, as there was no Spearman correlation between baseline dVA and dVA improvements (rho_(N=38)_ = 0.009, *p* = 0.96). Importantly, this main effect was significantly modified by predictors ‘group’ and ‘presence of astigmatism’, which indicated that astigmatism had a significant impact on interocular dVA improvements overall. The effect was also different between groups and in how baseline dVA affected improvement across groups (main effect of ‘presence of astigmatism’: F_(1,25)_ = 32.71, *p* < 0.0001; ‘group × presence of astigmatism’: F_(1,25)_ = 55.90, *p* < 0.0001; ‘baseline dVA × presence of astigmatism’: F_(1,25)_ = 20.53, *p* = 0.0001; ‘group x presence of astigmatism × baseline dVA’: F_(1,25)_ = 33.45, *p* < 0.0001; while main effect of ‘group’ and ‘group × baseline dVA’ interaction were not significant: all Fs ≤ 1.72, *p*s ≥ 0.20). The presence of astigmatism was a significant limiting factor for dVA improvements only in the pediatric group (Fig. 4d). Non-astigmatic children showed progressively more improvements as a function of baseline dVA (i.e. the worse the baseline dVA, the more the dVA improvement is; rho_(N=8)_ = 0.93, *p* = 0.0008), while surprisingly there was an opposite, i.e. negative correlation for astigmatic children (rho_(N=13)_ = − 0.84, *p* = 0.0003). Pediatric patients showing dVA improvements were non-astigmatic children with at least three dVA lines difference between the eyes and astigmatic children with two or less lines difference in dVA between the eyes at the baseline examination. On the other hand, adults did not improve regardless of their baseline dVA, or whether they had astigmatism in their amblyopic eye (Fig. 4e).

Baseline nVA had a significant effect on the amount of dVA improvement (main effect F_(1,25)_ = 10.05, *p* = 0.0040). Interestingly, nVA showed an opposite trend: the lower the interocular nVA, the higher the dVA gain provided by the training. This was confirmed by partial correlations, in which either nVA or dVA was controlled. There was a significant positive partial correlation between baseline dVA and dVA gain (r_partial (N=38)_ = 0.48, *p* = 0.002) and, on the contrary, there was a significant negative correlation between baseline nVA and dVA gain (r_partial (N=38)_ = − 0.53, *p* = 0.001). The effect of baseline nVA in the training outcomes was similar between groups regardless of astigmatism, hence these interactions were not accounted for in the final model. Thus, dVA gain, the main target of amblyopic treatments, was dependent on both dVA and nVA baseline values in an opposite manner, suggesting that moderate cases of amblyopia, considering interocular nVA and dVA, may be prognostic of dVA recovery following binocular treatment. Finally, two additional factors improved model fit substantially: etiology (F_(3,25)_ = 3.59, *p* = 0.028) and previous occlusion therapy (F_(1,25)_ = 4.49, *p* = 0.044). Even though there were no significant post-hoc differences among etiologies, anisometropic patients (A & AA) improved roughly twice as much as strabismic patients (S & SA), while patients with previous occlusion improved more, on average.

##### Interocular near visual acuity

The best prediction model for near visual acuity changes, with a fit of R_multiple_ = 0.92 and adjusted R^2^ = 0.79 (F_(11,27)_ = 13.78, *p* < 0.0001) explaining 79% of the variance, was found. Since baseline interocular nVA and dVA were closely correlated (rho_(N=42)_ = 0.77, p < 0.0001), similar models were used with a few modifications. In addition to the full factorial model of {‘baseline interocular nVA’, ‘group’, and ‘presence of astigmatism’}, the final model included ‘etiology’ and ‘sightedness’ as predictors. There were three patients classified as outliers (i.e. standard residual ≥ 2 SD), who were removed. The final prediction model included 39 patients. Baseline nVA had the strongest effect on nVA improvements (main effect: F_(1,27)_ = 40.26, *p* < 0.0001): the worse the nVA, the better the nVA improvement. Importantly, this general effect was significantly modified by predictors ‘group’ and ‘presence of astigmatism’, meaning that astigmatism had a significant effect overall, between groups and on how baseline nVA affected improvements (main effect of ‘presence of astigmatism’ F_(1,27)_ = 5.12, *p* = 0.032; ‘group × presence of astigmatism’: F_(1,27)_ = 5.82, *p* = 0.022; and ‘baseline nVA’ × ‘presence of astigmatism’: F_(1,27)_ = 9.78, *p* = 0.0042, while main effect of ‘group’, ‘group × baseline nVA’ interaction, and the three-way interaction between them were not significant all Fs ≤ 0.86, *p*s ≥ 0.36). In fact, astigmatism was a strong limiting factor for nVA improvements only in children (Fig. 5d): the majority of children with astigmatism either failed to improve or improved much less than expected based on their baseline interocular nVA (post-hoc astigmatic vs. non-astigmatic children *p* = 0.039). On the other hand, astigmatism had no effect on nVA improvements in adult patients (Fig. 5e; post-hoc astigmatic vs. non-astigmatic adults *p* = 0.63). This was confirmed by Spearman correlations: there was a significant positive correlation between baseline nVA and nVA gain for the adults (rho_(N=16)_ = 0.78, *p* = 0.0004), while there was no significant correlation for the children (rho_(N=23)_ = 0.33, *p* = 0.12). Importantly, however, the latter was explained by the presence of astigmatism: when astigmatic children were excluded from correlation, it became significant (rho_(N=8)_ = 0.91, *p* = 0.002). The few astigmatic children, who were the exception to the above ‘rule’, almost exclusively had pure astigmatism without spherical refractive error.

Finally, two additional factors played significant roles in explaining the amount of interocular nVA improvements: sightedness and etiology. Hypermetropia (far-sightedness) was predictive of a more robust nVA improvement especially in the adult population. Far-sighted patients, whose vision could have been compromised for near vision before they received correction, improved significantly more than near-sighted (i.e. myopic) patients (F_(1,27)_ = 17.42, *p* = 0.0003), even though there was no baseline difference between the groups either in amblyopic or interocular nVA. This emphasizes the potential use of binocular approaches to treat adult far-sighted patients, who are overaged for standard monocular (patching) treatments. Etiology also had a significant effect on nVA improvement (F_(3,27)_ = 6.49, *p* = 0.0019). Subjects, who had both spherical and astigmatic anisometropia (AA) gained significantly less compared with all other etiology groups (post-hoc p = 0.0003, p = 0.0005, and p = 0.093 for AA vs. A, AA vs. SA, and AA vs. S, respectively).

#### Contrast sensitivity deficits can be treated above a critical visual acuity in the presence of stereopsis

Interocular contrast sensitivity (CS) changes were reliably predicted with a model fit of R_multiple_ = 0.93 and adjusted R^2^ = 0.81 (F_(10,27)_ = 16.47, *p* < 0.0001) explaining 81% of the variance. The final and most economical model included ‘baseline interocular CS’, ‘age-group’ (i.e., < 9 years, 10–19 years, 20–39 years, and > 40 years), ‘baseline interocular CS × age-group’ interaction, ‘measurable stereopsis at baseline’, ‘post-treatment poor dVA (≥ 0.4 logMAR) in the amblyopic eye’, ‘measurable stereopsis × poor amblyopic dVA’ interaction as predictors. The final prediction model included 38 patients, as four patients were classified as outliers (i.e. standard residual ≥ 2 SD) and were removed. Baseline CS had the strongest effect on CS improvements (main effect: F_(1,27)_ = 79.03, *p* < 0.0001): the worse the CS, the better the CS improvement (Fig. 6d). Importantly, age-group also had a significant effect on the therapeutic outcomes (main effect: F_(3,27)_ = 3.89, *p* = 0.020) and a significant interaction with baseline CS (F_(3,27)_ = 5.71, *p* = 0.0037): the 20–39 years age group showed the least overall improvement, which was significantly different from the larger improvement of the < 10 years child age group (post-hoc *p* = 0.038). Moreover, as the interaction indicated, the correlation between baseline CS and CS improvements was age-group dependent. In the 20–39 years age group, the individual CS improvement was independent of baseline CS. On the other hand, there were consistent dependencies in the other groups. This was corroborated by Spearman correlations: there were significant positive correlations between baseline CS and CS improvements in the < 10 years, 10–19 years, and > 40 years groups (rho_(N=11)_ = 0.65, *p* = 0.032, rho_(N=12)_ = 0.82, *p* = 0.0012, and rho_(N=5)_ = 1.00, *p* = 0.017, respectively), while it was not significant in the 20–39 years age group (rho_(N=10)_ = − 0.15, *p* = 0.67). Most notable was this dependency in the 10–19 years age group, in which data points fell on a relatively straight line, therefore, close of completely resolving baseline CS deficits in patients who are generally regarded as too old to be treated.

Importantly, however, two factors limiting CS gain were found: poor amblyopic dVA at the end of the treatment, and non-measurable stereopsis at the baseline examination. The criterion for poor dVA was ≥ 0.4 logMAR (≤ 20/50 Snellen acuity), the corresponding Snellen equivalent to the highest contrast stimulus with 12 cpd grating, which was the lowest spatial frequency where most of the patients had worse CS in the amblyopic compare with the fellow eye. Thus, the amblyopic dVA achieved was converted into a binary predictor indicating whether dVA better than the limiting 0.4 has been achieved. While this factor does not have any predictive value in the classical sense as it can only be obtained as an outcome of the training, the inability to resolve the spatial frequencies demonstrating amblyopic CS deficit does explain the lack of improvement. Significant effects for both predictors (F_(1,27)_ = 6.92, *p* = 0.014 and F_(1,27)_ = 16.28, *p* = 0.0004 for ‘poor dVA’ and ‘measurable stereopsis’, respectively), and, more importantly, a significant interaction between them (F_(1,27)_ = 16.38, *p* = 0.0004) were observed. While both poor baseline amblyopic dVA and non-measurable stereopsis hindered CS improvement, it was the combination of the two factors that prevented CS gain (Fig. 6e). Thus, measurable stereopsis and relatively preserved amblyopic dVA were required for recovering amblyopic CS regardless of age. In fact, most patients in this category showed improved amblyopic CS: 20 out of 24 patients achieved CS within the normal range defined for the SWCT test. Therefore, fully treated.

## Discussion

The results showed that the 3D augmented reality interactive game training reported here was able to improve visual functions in both pediatric and adult patients with amblyopia, which could be predicted considering baseline clinical parameters. The training successfully improved stereoacuity in amblyopic patients. Moreover, significant monocular improvements in near visual acuity and contrast sensitivity were observed even if amblyopic changes were corrected for possible learning effects (i.e. normalized to the changes observed in the dominant eye). Importantly, critical factors strongly limited or even prevented improvements: (1) astigmatism in children limited visual acuity improvement both at near and distance, (2) comparable fixation stability between the eyes was necessary for stereopsis recovery in stereoblind patients, and finally, (3) stereopsis and a critical minimal visual acuity was required for contrast sensitivity improvements. This is the first report, to our knowledge, demonstrating patterns of predictive values in clinical parameters other than the isolated baseline values to estimate treatment effectiveness. Based on these results, we propose a unified treatment protocol.

In the present investigation, binocular training induced interocular change in near visual acuity (nVA) was assessed and proved to be more sensitive to treatment modulation compared with distance visual acuity (dVA), which did not show substantial improvement after interocular normalization. This is consistent with pediatric clinical experience that improvement in nVA precedes that of dVA and underlines the importance of measuring nVA when assessing the effectiveness of occlusion therapy in children. However, nVA improvements were surprisingly more evident in adult compared with pediatric patients, likely because of the presence of astigmatism in 60% of the children, which was observed to be a limiting factor for nVA improvements in the pediatric group. In fact, astigmatism in children strongly limited VA improvements in general: only astigmatic patients with very mild amblyopia showed VA improvements. Interestingly, while astigmatism in the amblyopic eye had little effect on the adult’s nVA improvement, patients with both spherical and astigmatic anisometropia had less chance of improving regardless of age. These results are in line with findings from Hussein et al.^49^, who reported clinical factors limiting the success of occlusion therapy in a retrospective study and have also confirmed that children with astigmatism (≥ 1.5D) were unlikely to achieve the desired outcomes. Most studies evaluating the relationship between astigmatism and amblyopia were conducted retrospectively focusing on the orientation of the astigmatic meridian^68–70^. In fact, astigmatic children are at risk for visual dysfunctions^71^. For instance, young infants at about 6 months old show lower grating visual acuity with proper astigmatic correction compared with non-astigmatic children^72^. Moreover, mild to moderate amblyopic children with astigmatism have significantly worse stereoacuity compared with hyperopic or myopic patients without astigmatism^73^. Even though large astigmatic refraction errors, especially in anisometropic patients, can be a challenge to reliably measure in children^74^, it would be crucial for astigmatic children to receive the best correction as this alone could improve visual functions over time^75^. Unfortunately, not enough emphasis is given to astigmatism when prescribing optical correction in children, which may contribute to its limiting effect on visual improvements in amblyopic children.

Foveal fixation depends on a diversity of voluntary and involuntary eye movements and eccentric (extra-foveal) fixation is closely associated with long-term visual acuity decrease after amblyopic treatment^76^. It has been previously established that (1) poor fixation stability is associated with poor monocular and binocular functions in amblyopic patients^16–18,77–79^; (2) the time needed for the recovery and stabilization of visual acuity may be shorter for patients with better fixational abilities of the amblyopic eye in occlusion therapy^56–58^; and (3) fixation stability can be improved in childhood^80^ and even in adult patients over the critical period of development^81–85^. This is the first report, to our knowledge, showing that the more similar the fixation stability between the eyes, the more likely a patient classified as stereoblind according to Titmus test is to develop a certain level of stereopsis as a result of binocular treatment, regardless of etiology or severity of amblyopia and, more importantly, regardless of age. Supporting this, fixational eye movements abnormalities, i.e. fusion maldevelopment nystagmus syndrome (FMNS) and nystagmus without FMNS have also been found to prevent and limit stereopsis improvements, respectively^57^, with no difference in the stereoacuity gain among amblyopic etiologies. Taken together previous reports and the present data, future investigations may consider proper fixation stability as a clinical requirement for visual improvements. The present results might also be potentially relevant for visual scientists and clinicians planning and designing future study protocols for the upcoming clinical trials to treat amblyopia^26^.

Visual acuity is still the standard clinical parameter for characterizing amblyopic status and for the management of several diseases affecting the visual system, even though it has been extensively reported that luminance contrast sensitivity (CS) is highly related to the quality of vision^86^. Moreover, CS provides a more complete picture of spatial vision compared to visual acuity, besides its potential to measure binocular balance in amblyopic patients^3,64,87^. In line with our results, significant contrast sensitivity improvements have been reported in adult amblyopic eyes following dichoptic training^44^ and perceptual learning^45,46^. Here we have further demonstrated that CS improvements can be achieved regardless of age or amblyopic etiologies, but only if at least a coarse stereovision or a minimum amblyopic visual acuity is present (≤ 0.4 logMAR), to allow for reading the higher frequencies of the SWCT chart. Importantly, our results also show, that in the absence of the above limiting factors, almost complete CS recovery is possible with the binocular approach reported here. In teenage patients (age 10–19 years), the slope of the regression line between baseline interocular CS and change in interocular CS was close to − 1 (β_1_ = − 0.79), resulting in interocular CS difference of less than 10% of that of the dominant eye. As a matter of fact, 83% of patients without limiting factors for CS had achieved amblyopic CS that was in the normal range.

The present study utilized a new binocular method focused on stereo image presentation in an immersive 3D AR environment, similar to which only a handful of studies have pioneered so far^39,43^. Thus, it was conducted as a critical first step of exploring and proving its potential on a patient population moderate in size. This inherently holds some limitations to our study. First, it is unclear whether our findings would be completely generalizable to longitudinal studies in a larger group of patients or to dichoptic treatment approaches already undergone clinical investigation. Second, the study design did not include a control (non-treated/occluded) group of amblyopic patients, which could have been used to evaluate the effectiveness of the present training. Nevertheless, by calculating interocular values in the present study, amblyopic improvements were normalized to that of the dominant eye, considering the decrease in interocular values over time. Such a decrease is not expected to arise from a simple learning effect or the test–retest variability of the conducted tests, as ETDRS VA test–retest variability is comparable for amblyopic, fellow, and control eyes^88^. However, the evaluation of stereoacuity improvements could have been influenced by learning or test–retest variability. Therefore, these findings require further confirmation with a larger group of patients including treated and control groups, especially given that randomized clinical trials have so far failed to prove dichoptic treatments superior to control treatments^26,60,89–91^.

**Figure 7:**
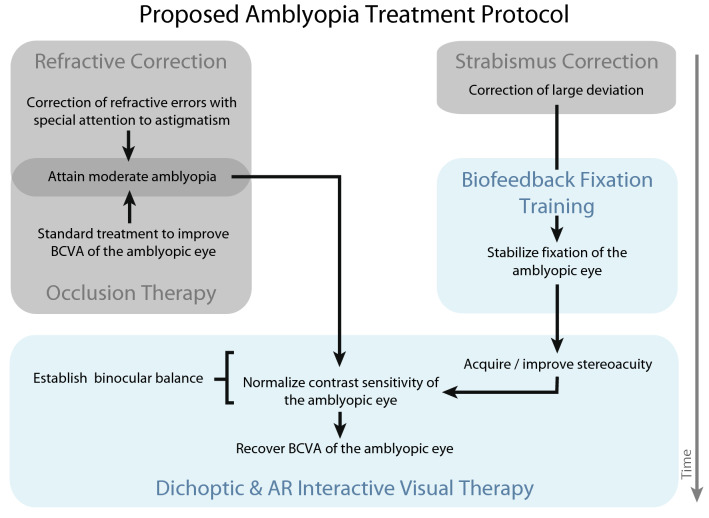
Proposed amblyopia treatment protocol for restoring binocular balance and alleviating amblyopia. Gray patches correspond to standard-of-care treatment of amblyopia, while blue patches correspond to new, emerging alternatives of amblyopia therapy. Application of each treatment modality is based on individual patient profile, laying the grounds for personalized treatment. Black arrows indicate subsequent steps, while gray arrow represents time.

Taken together, the present results emphasize the benefits of 3D binocular training in the management of amblyopia: significant, lasting improvements of monocular and binocular vision in both pediatric and adult patients, supporting its efficacy even after the critical period of visual development. Moreover, the findings shed light on specific clinical parameters that may help to anticipate the magnitude of visual improvements induced by binocular treatments, which may contribute to a better understanding of monocular/binocular interactions following binocular training. Figure 7 shows a meaningful integration of the different existing therapeutic approaches into a combined treatment protocol for amblyopia based on our results. In practice, best refractive correction is provided with attention to the proper correction of the astigmatism^92^, especially for young children, as this has been demonstrated here and elsewhere^49^ that larger astigmatism can be a serious limiting factor in visual acuity improvement during occlusion and dichoptic therapies. After visual acuity improvement gained from optical correction has plateaued, occlusion therapy has its place if amblyopic visual acuity is still lower than 0.4 logMAR, or the child is too young to be treated using dichoptic games. Meanwhile, if significant fixation instability of the amblyopic eye is observed, the treatment should target fixation stability balance between the eyes, which was shown here to be required for stereovision recovery in the case of stereoblind patients. After the best possible fixation stability is achieved, a treatment scheme can commence involving both binocular (i.e. stereo) and dichoptic (2D complementary images) stimulation in an interactive and engaging format to provide better stereoacuity or at least coarse stereopsis and robust visual acuity improvement, respectively. When amblyopic visual acuity has achieved a mild-moderate range and stable stereovision is measured, contrast sensitivity improvement or even normalization could be expected. Lastly, with binocular functions and interocular balance restored, full and lasting visual acuity recovery in amblyopia may be attained with further treatment^40^. This protocol may help clinicians to recommend therapeutic solutions for a personalized and more reliable visual restoration in amblyopia.

## Supplementary Information

Supplementary Information.
