# Publisher Correction: Fixation instability, astigmatism, and lack of stereopsis as factors impeding recovery of binocular balance in amblyopia following binocular therapy

**DOI:** 10.1038/s41598-022-17574-5

**Published:** 2022-08-02

**Authors:** Éva M. Bankó, Mirella Telles Salgueiro Barboni, Katalin Markó, Judit Körtvélyes, János Németh, Zoltán Zs. Nagy, Zoltán Vidnyánszky

**Affiliations:** 1 Brain Imaging Centre, Research Centre for Natural Sciences, Magyar Tudósok Körútja 2, Budapest, 1117 Hungary; 2 Department of Ophthalmology, Semmelweis University, Budapest, Hungary; 3 Bionic Innovation Center, Budapest, Hungary

Correction to: *Scientific Reports* https://doi.org/10.1038/s41598-022-13947-y, published online 20 June 2022

The original version of this Article contained an error in Figure 6 where the axes labels were omitted in the subplots. The original Figure 6 and accompanying legend appear below.

**Figure 6:**
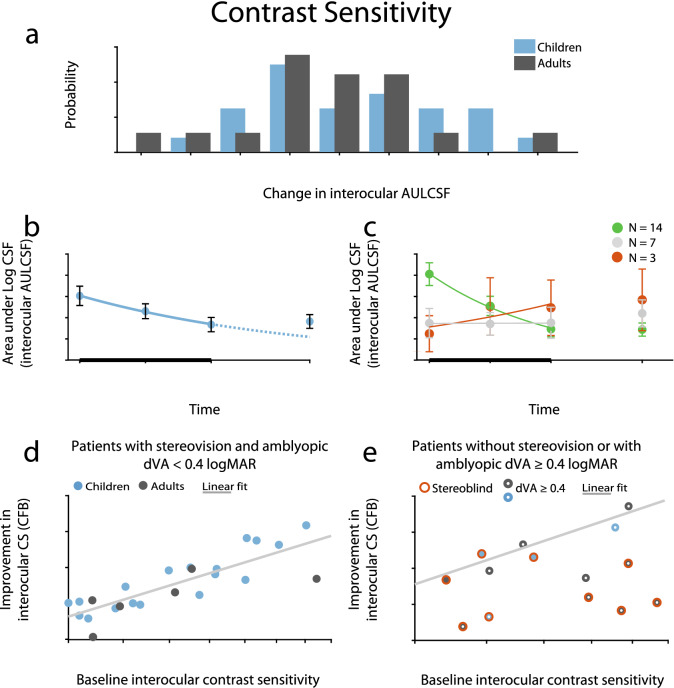
Improvement in AULCSF (Area Under Log Contrast Sensitivity Function). (**a**) Distribution of change from baseline (CFB) in AULCSF in children and adults. (**b**) Time course of contrast sensitivity recovery in children. The negative exponential fit indicates improvement (solid line) during the therapy (thick black line) and the expected improvement rate (dotted line) had the therapy been continued. Children’s improvement remained stable at 1-m follow-up without regression. (**c**) Division of children’s change over time based on the exponent of the fitted exponential: green, red, and gray indicates improvement, decline, and no change, respectively. (**d**, **e**) Results of the prediction analysis: (**d**) Patients’ improvement had a definitive dependence on their baseline contrast sensitivity: patients improved almost as much as there was room for improvement, given that they did not have either of two limiting factors: initial stereoblindness or acuity equal to or below a critical visual acuity of 0.4 logMAR. (**e**) The improvement of patients with only one of the limiting factors also lie along the same linear fit calculated for patients without either of the limiting factors, whereas patient affected by both factors showed no or very limited improvement. Red circles signify initially stereoblind patients, white dots in the center of datapoints mark acuity equal to or below 0.4 logMAR, while a light gray line indicates a linear fit for patients without limiting factors, which is copied to (**e**) as well. N_Ch_ = 24, N_Ad_ = 18, except for (**d**, **e**), where N_Ch_ = 22, N_Ad_ = 16. Means ± SEM.

The original Article has been corrected.

