# Supplementary material for: Fixation instability, astigmatism, and lack of stereopsis as factors impeding recovery of binocular balance in amblyopia following binocular therapy
Source: Sci Rep. 2022 Jun 20;12:10311. doi: 10.1038/s41598-022-13947-y (PMC9209502; doi:10.1038/s41598-022-13947-y)
Supplement: Supplementary file 1 — Supplementary Information. [file 41598_2022_13947_MOESM1_ESM.pdf]

SUPPLEMENTARY METHODS & RESULTS

Fixation instability, astigmatism, and lack of stereopsis as factors impeding recovery of binocular balance in amblyopia following binocular therapy

Éva M. Bankó, Mirella Telles Salgueiro Barboni, Katalin E. Markó, Judit Körtvélyes, János Németh, Zoltán Zs. Nagy, Zoltán Vidnyánszky

Bivariate contour ellipse area to quantify fixation stability

Fixation stability was also quantified by calculating the bivariate contour ellipse area (BCEA) in Matlab (Mathworks, Natick, MA) using the formula:

$$BCEA = 2k\pi\sigma_H\sigma_V\sqrt{1-\rho^2} \quad ,$$

where σ_H and σ_V is the standard deviation of fixation position in the horizontal and vertical meridian, respectively, and ρ is the product-moment correlation of these two components. The constant k was chosen to be 1.14 to encompass 68% of fixation points based on the following probability function:

$$P = 1 - e^{-k}.$$

Fixation stability of the amblyopic eye expressed as BCEA showed strong correlation with the median Euclidian distance measure used in the main analysis (Fig.S1a; Spearman $rho_{(N=19)} = 0.96$, $p < 0.0001$ and $rho_{(N=13)} = 0.85$, $p = 0.0003$, for children and adults, respectively). The same was true for the interocular difference of these two measures, which was used as a predictor in the prediction analysis of stereoacuity gain (Fig.S1b; $rho_{(N=19)} = 0.72$, $p = 0.0006$ and $rho_{(N=13)} = 0.73$, $p = 0.0046$, for children and adults, respectively).

The model used for predicting stereovision improvement was the same as applied for median distance with similar results. Model fit was $R_{multiple} = 0.87$ and adjusted $R_2 = 0.74$ ($F_{(3,28)} = 30.49$, $p < 0.0001$) explaining 74% of the variance of the data (N=32). Baseline stereoacuity had the strongest effect on stereoacuity improvement ($F_{(1,28)} = 89.84$, $p < 0.0001$): the worse the stereoacuity was for a given patient, the more that patient could improve, indicating that the training strongly modulated stereoacuity. Importantly, relative baseline fixation stability had a significant effect on therapy outcome ($F_{(1,28)} = 4.63$, $p = 0.040$) as well as a significant interaction with baseline stereoacuity ($F_{(1,28)} = 4.76$, $p = 0.038$): patients with better interocular fixation stability (i.e. smaller difference in fixation between the eyes) had a higher potential for stereoacuity improvement. However, as the interaction indicated, this effect was dependent on baseline stereoacuity: interocular fixation stability was a strong predictor for patients with only coarse or nil stereoacuity (≥3500"; Fig. S1c).

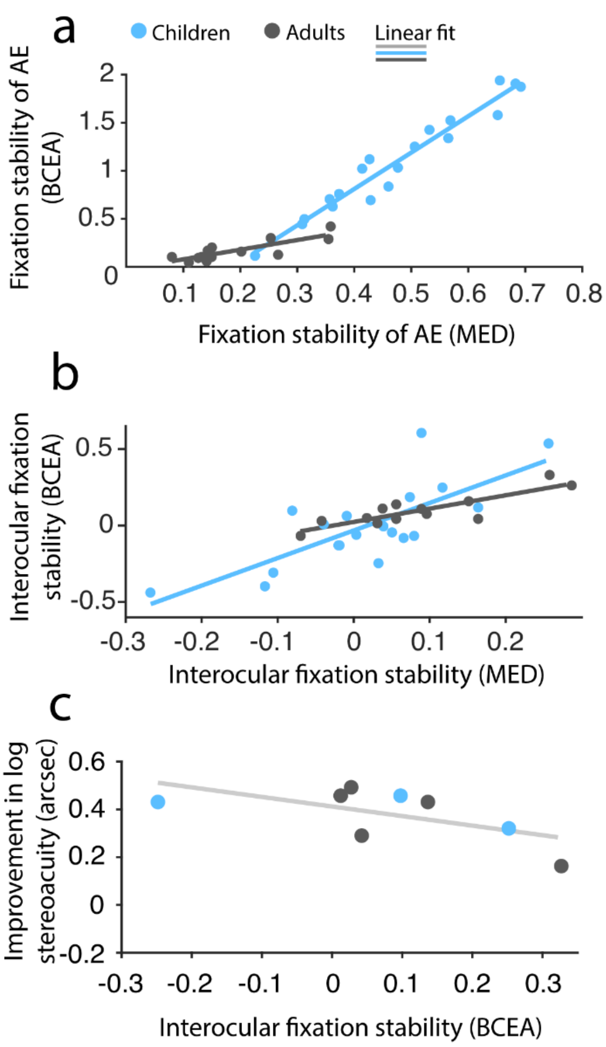

Supplementary Figure S1. Fixation stability calculated as the bivariate contour ellipse area (BCEA). (a-b) *Correlation between BCEA and median Euclidean distance (MED)* for the amblyopic eye (a) and for the interocular difference (b). (c)

Improvement of stereoblind or patients with crude stereoacuity (≥3500") strongly depended on their relative fixation stability.

Induced changes in monocular visual functions

Analyzing the pediatric population, significant improvement from baseline at the 20h visit (V_{20h}) was found in all the monocular visual functions of the amblyopic eye (Fig. 2; Wilcoxon matched pairs test: Z = 4.11, p < 0.0001, Z = 3.92, p < 0.0001, and paired t- test: t(23) = -6.98, p < 0.0001 for distance VA (dVA), near VA (nVA), and contrast sensitivity (CS), respectively). These changes proved to be lasting within the one month follow-up period without any form of treatment, because there was no significant difference between values measured at V_{20h} and at the follow-up visit (V_{FU}) (both |t(19)| ≤ 0.63 and p ≥ 0.54 for dVA and CS; Z = 1.21, p = 0.22 for nVA). Surprisingly, the monocular visual functions of the dominant eye also showed significant, albeit less pronounced, improvement as a result of the training in the cases of dVA and CS (Fig. 2; t(23) = 5.51, p < 0.0001, Z = 4.18, p < 0.0001), whereas the change in the dominant eye's nVA did not reach the Bonferroni-corrected significance threshold (p < 0.05/6 = 0.008; Z = 2.04, p = 0.041).

The pattern of amblyopic improvements was similar in the adult population: all amblyopic visual functions improved significantly (Fig. 2; Z = 2.94, p = 0.0033, t(17) = 3.99, p = 0.0010, and t(17) = 4.51, p = 0.0003 for dVA, nVA, and CS, respectively). However, the change in dominant eye functions altogether failed to reach the Bonferroni corrected threshold of significance (t(17) = 2.46, p = 0.025; Z = 0.71, p = 0.48; and t(17) = 2.26, p = 0.037 for dVA, nVA, and CS, respectively).

Direct comparison of the difference between post- and pre-treatment measurements (i.e. CFB: change from baseline) across groups for all monocular visual functions showed that the two groups were affected similarly by the treatment: the difference in means or median values of the visual functions between groups did not reach the Bonferroni corrected significance threshold (p=0,008) in any of the measured functions either in the amblyopic eye (two-samples t-test t(40) = 2.19, p = 0.033, Mann-Whitney U-test (n1 = 24, n2 = 18): Z = -0.625, p = 0.51, and t(40) = 2.15, p = 0.038 for dVA, nVA, and CS, respectively), or for the dominant eye (t(40) = 1.02, p = 0.32, t(40) = 1.85, p = 0.072, and t(40) = 1.54, p = 0.13 for dVA, nVA, and CS, respectively).

The overall pattern of positive changes in the dominant eye raises the possibility that task-related learning effects and test-retest variability has contributed to the observed improvements, the effects of which are not expected to differ between eyes. Therefore, we focused our analyses on interocular differences in case of VA and CS as a means of normalizing to changes not specific to the binocular training used.

Factors limiting improvement in monocular visual functions of the amblyopic eye

We have also analyzed the improvement of the amblyopic eye alone to investigate whether the factors we found limiting the rebalancing of the interocular difference were also valid in the case of the progress in visual functions of the amblyopic eye. The same ANCOVA models were used here, outliers, however, were based on the current models.

Astigmatism has an effect only on distance visual acuity gain in children

Distance visual acuity. The final prediction model fit for distance visual acuity was $R_{multiple}$ = 0.80 and adjusted R^2 = 0.48 ($F_{(12,27)}$ = 4.01, p = 0.0013), explaining 48% of data variance and, similarly to the one used for interocular values, included the full factorial model of {'baseline distance visual acuity of AE (dVA)', 'group', and 'presence of astigmatism'}, and 'baseline near visual acuity of AE (nVA)', 'etiology', and 'past occlusion' as predictors. However, there were only two patients whose data were classified as outliers (i.e. standard residual ≥ 2 SD), and were removed from the final model, leaving 40 patients for the prediction model. As opposed to the model with interocular values, baseline dVA had the most pronounced effect on therapy outcome (main effect: $F_{(1,27)}$ = 14.37, p = 0.0008). This could not be attributed to a simple tendency of patients with higher interocular difference in dVA being able to improve more, as there was no significant Spearman correlation between baseline dVA and dVA improvement ($rho_{(N=40)}$ = -0.21, p = 0.20). Importantly, this main effect was modified by predictors 'group' and 'presence of astigmatism',

indicating that the presence of astigmatism had a significant impact on dVA improvement overall, between groups and on how baseline dVA affected improvement across groups ('presence of astigmatism' main effect: $F_{(1,27)} = 5.37$, $p = 0.029$; 'group × presence of astigmatism': $F_{(1,27)} = 4.26$, $p = 0.049$; 'baseline dVA × presence of astigmatism': $F_{(1,27)} = 5.09$, $p = 0.032$; and the three-way interaction between these variables: $F_{(1,27)} = 5.56$, $p = 0.026$; while main effect of 'group' and 'group × baseline dVA' interaction were not significant: all Fs ≤ 0.77, *p*s ≥ 0.39). The presence of astigmatism was an important limiting factor on dVA improvement only in children (Fig. S2a.). Non-astigmatic children showed progressively more improvements as a function of baseline dVA (i.e. the worse the baseline dVA, the more the dVA improvement is; $rho_{(N=9)} = 0.74$, $p = 0.023$), while surprisingly there was an opposite, i.e. negative correlation for astigmatic children ($rho_{(N=13)} = -0.83$, $p = 0.0005$). Pediatric patients showing dVA improvements were non-astigmatic children with a baseline vision of ≥0.3 logMAR, and astigmatic children with a baseline vision of ≤0.3 logMAR at distance. On the other hand, adults did not improve regardless of their baseline dVA, or whether they had astigmatism in their amblyopic eye (Fig. S2b).

Baseline nVA also had a significant effect on the amount of dVA improvement achieved by the amblyopic eye ($F_{(1,27)} = 13.01$, $p = 0.0012$), showing an opposite trend to that of dVA, that is the better the amblyopic baseline nVA, the higher the gain in dVA could possibly be, which was also visible in Spearman correlation analysis ($rho_{(N=40)} = -0.49$, $p = 0.0013$). Contrary to the model with interocular values, etiology (main effect: $F_{(3,27)} = 0.58$, $p = 0.63$) and the history of occlusion therapy (main effect: $F_{(1,27)} = 1.11$, $p = 0.30$) did not have significant effects on dVA gain of the amblyopic eye.

Near visual acuity. Using the previously obtained best prediction model for interocular near visual acuity change gave a model fit of $R_{multiple} = 0.75$ only and adjusted $R^2 = 0.39$ ($F_{(11,28)} = 3.31$, $p = 0.0051$), explaining only 39% of the data variance. In addition to the full factorial model of 'baseline near visual acuity of AE (nVA)', 'group', and 'presence of astigmatism', the model included 'etiology' and 'sightedness' as predictors. There were two patients whose data were classified as outliers (i.e. standard residual ≥ 2 SD), and were removed from the final model, leaving 40 patients for the prediction model. Baseline near VA had the only significant effect on nVA improvement ($F_{(1,28)} = 12.87$, $p = 0.0013$): the worse the nVA of the amblyopic eye was for a given patient, the more that patient could improve. The modification of this general effect by predictors 'group' and 'presence of astigmatism' were not significant. The only interaction that came anywhere close to being significant was the interaction between 'group × baseline nVA of AE' ($F_{(1,28)} = 2.35$, $p = 0.14$; while all Fs ≤ 0.61, *p*s ≥ 0.44 for other interactions and main effects of either 'group' or 'presence of astigmatism'). When looking at Spearman correlations, however, there was a clear difference between groups, how the nVA gain depended on the baseline values: children's improvement showed no dependence (Fig. S2c; $rho_{(N=24)} = 0.26$, $p = 0.22$), while there was a significant positive correlation between baseline nVA of the amblyopic eye and its gain in nVA (Fig. S2d; $rho_{(N=16)} = 0.69$, $p = 0.003$). Even when astigmatism was excluded in children, no correlation has emerged, much to the contrary of the interocular results. However, the etiological difference in nVA improvement of the amblyopic eye looked similar to the results obtained with interocular values: on average, subjects, who had both spherical and astigmatic anisometropia (AA) gained the least from this treatment compared with all other etiology groups. Nonetheless, this main effect did not reach the significance threshold ($F_{(3,28)} = 2.68$, $p = 0.066$), just as 'sightedness' had no significant effect on nVA gain of the amblyopic eye alone ($F_{(1,28)} = 0.18$, $p = 0.67$).

In conclusion, the results predicting the visual acuity changes of the amblyopic eye alone have showed tendencies that resembled to the prediction results obtained with interocular, i.e. normalized values, although they presented a much less clear picture, pointing to baseline VA influencing more (if not being the only strong predictor) of VA improvements, as it is usually reported and typically considered during amblyopic treatment.

Contrast sensitivity deficit is capable of resolving above a critical acuity in the presence of stereopsis

Contrast sensitivity change of the amblyopic eye was the only monocular measure that could be predicted

with a sufficient reliability with a model fit of $R_{multiple} = 0.84$ and adjusted $R^2 = 0.62$ ($F_{(10,31)} = 7.60$, $p < 0.0001$), explaining 62% of the data variance. The final model included ‘baseline contrast sensitivity (CS) of AE’, ‘age-group’ (i.e. <9y, 10-19y, 20-39y, and >40y), ‘baseline

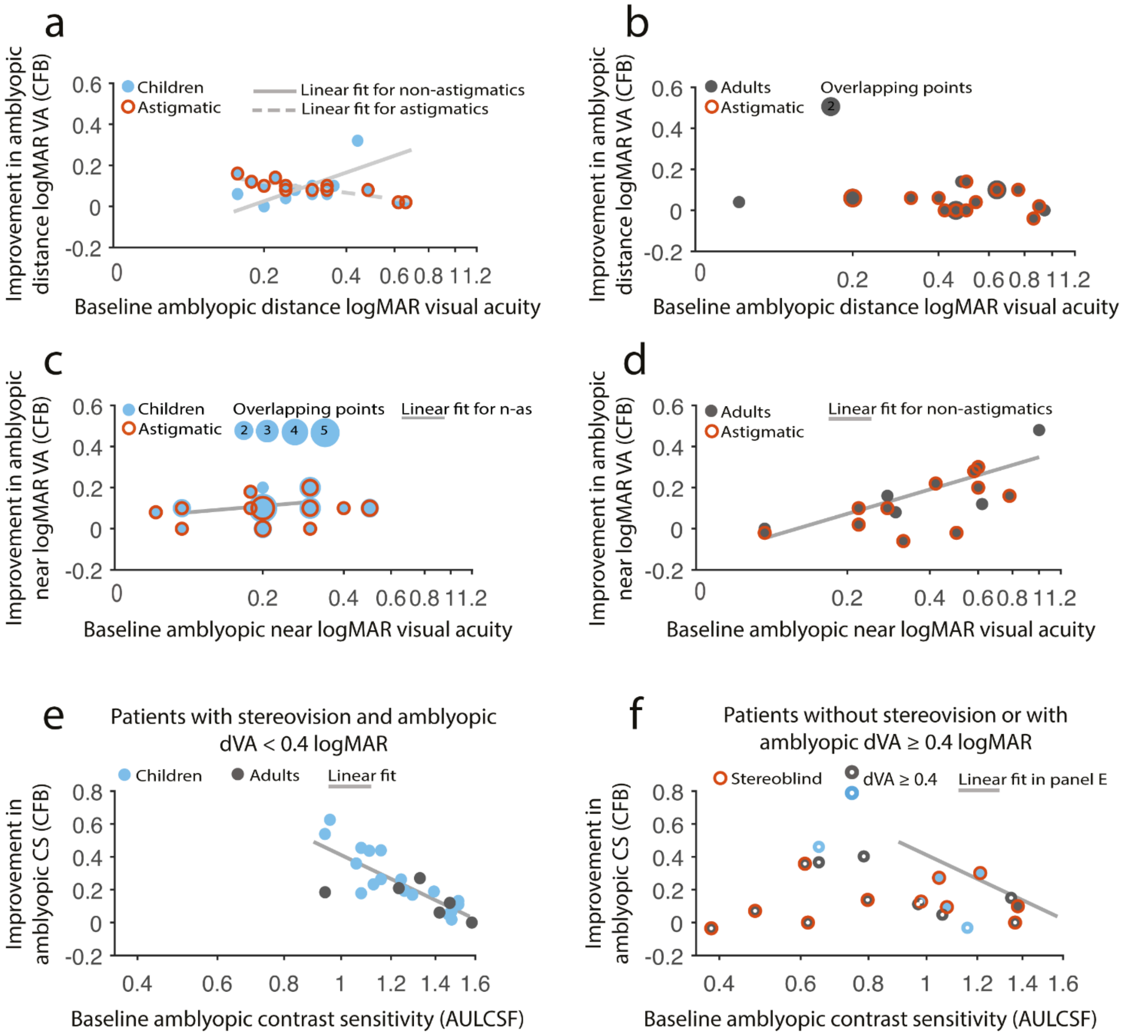

Supplementary Figure S2. Results of the prediction models for monocular amblyopic visual functions. (a-b) *Amblyopic distance visual acuity change.* (a) Children’s amblyopic dVA recovery strongly depended on the presence of astigmatism: non-astigmatic children’s potential for improvement increased with worse baseline amblyopic dVA, while the opposite was true for astigmatic children. (b) Adults did not improve regardless of their baseline dVA, or whether they had astigmatism in their amblyopic eye. N_{Ch}=22, N_{Ad}=18. (C-D) *Amblyopic near visual acuity change.* (c) The limiting effect of astigmatism was not evident in children’s amblyopic nVA recovery, which seemed independent both of baseline amblyopic nVA and the presence of astigmatism. (d) Adults’ progress, on the other hand, depended on their baseline nVA, but was not impacted by astigmatism. N_{Ch}=24, N_{Ad}=16. Red circles signify astigmatic patients, light gray solid line indicates a linear fit for non-astigmatic children, while the dashed line is the linear fit for astigmatic children. (e-f) *Amblyopic contrast sensitivity change.* (e) Patients’ improvement had a definitive dependence on their baseline contrast sensitivity: the worse it was the more patients improved, given that they did not have either of two limiting factors: initial stereoblindness or acuity below a critical visual acuity of 0.4 logMAR. (f) However, patients with either one of the limiting factors showed no or very limited improvement. N_{Ch}=24, N_{Ad}=18. Red circles signify initially stereoblind patients, white dots in the center of data points mark acuity equal to or below 0.4 logMAR, while the light gray line indicates a linear fit for patients without limiting factors, which is copied to panel f as well.

interocular CS × age-group' interaction, 'measurable stereopsis at baseline', 'post-treatment poor dVA (≥0.4 logMAR) in the amblyopic eye', 'measurable stereopsis × poor amblyopic dVA' interaction as predictors There were no patients whose data had to be classified as outliers (i.e. standard residual ≥ 2 SD), thus all 42 patients were included in the prediction model. Baseline contrast sensitivity had the strongest effect on CS improvement (main effect: $F_{(1,31)} = 50.78$, $p < 0.0001$): the worse the CS, the better the CS improvement (Fig. S2e). Importantly, age-group also had a significant effect on therapy outcome (main effect: $F_{(3,31)} = 11.34$, $p < 0.0001$) as well as a significant interaction with baseline contrast sensitivity ($F_{(3,31)} = 8.91$, $p = 0.0002$): the 20-39y age group showed the least overall improvement, which was significantly different from the larger improvement of the <10y and 11-19y child age-groups (post-hoc $p = 0.022$ and $p = 0.0060$, respectively). Moreover, as the interaction indicated, the relationship between CS improvement and baseline CS was also age-group dependent. In the 10-19y age group, the individual CS improvement was highly dependent of baseline CS values. On the other hand, there were no clear dependencies on baseline CS in the other groups. This was corroborated by computing Spearman correlations. There was a significant negative correlation between baseline contrast sensitivity and CS improvement in the 10-19y age-group (Spearman $rho_{(N=12)} = -0.89$, $p = 0.0001$, while there were no such significant dependencies in the cases of the <10y, 20-39y, and >40y age groups ($rho_{(N=13)} = -0.39$, $p = 0.19$; $rho_{(N=11)} = 0.13$, $p = 0.71$, and $rho_{(N=6)} = -0.43$, $p = 0.40$, respectively). This dependency in the case of the 10-19y age group, was very strong in the amblyopic eye data as well: data points fell on a relatively straight line, therefore, close of completely resolving baseline CS deficits in patients who are generally regarded as too old to be treated.

Furthermore, the two limiting factors found previously for the interocular data – poor amblyopic distance VA (≥0.4 logMAR) on finishing the treatment, and the lack of stereopsis at baseline – were also shown to seriously compromise the CS gain of the amblyopic eye as a result of the treatment (Fig. S2f). Our results showed significant effects for both of the above predictors (main effects: $F_{(1,31)} = 7.50$, $p = 0.010$ and $F_{(1,31)} = 4.19$, $p = 0.049$ for 'poor dVA' and 'presence of stereopsis', respectively), but no interaction between them ($F_{(1,31)} = 1.10$, $p = 0.31$). Thus, the results of the prediction model obtained with the amblyopic eye alone is consistent with the interocular results: patients both with measureable stereopsis and with sufficient amblyopic distance VA are capable of recovering their amblyopic contrast sensitivity regardless of age (Fig. S2e).
